# Bioinspired Technologies to Connect Musculoskeletal Mechanobiology to the Person for Training and Rehabilitation

**DOI:** 10.3389/fncom.2017.00096

**Published:** 2017-10-18

**Authors:** Claudio Pizzolato, David G. Lloyd, Rod S. Barrett, Jill L. Cook, Ming H. Zheng, Thor F. Besier, David J. Saxby

**Affiliations:** ^1^School of Allied Health Sciences, Griffith University, Gold Coast, QLD, Australia; ^2^Gold Coast Orthopaedic Research and Education Alliance, Menzies Health Institute Queensland, Griffith University, Gold Coast, QLD, Australia; ^3^La Trobe Sport and Exercise Medicine Research Centre, La Trobe University, Melbourne, VIC, Australia; ^4^Centre for Orthopaedic Translational Research, School of Surgery, University of Western Australia, Nedlands, WA, Australia; ^5^Auckland Bioengineering Institute and Department of Engineering Science, University of Auckland, Auckland, New Zealand

**Keywords:** biomechanics, mechanobiology, wearable devices, tissue strain, biofeedback, modeling

## Abstract

Musculoskeletal tissues respond to optimal mechanical signals (e.g., strains) through anabolic adaptations, while mechanical signals above and below optimal levels cause tissue catabolism. If an individual's physical behavior could be altered to generate optimal mechanical signaling to musculoskeletal tissues, then targeted strengthening and/or repair would be possible. We propose new bioinspired technologies to provide real-time biofeedback of relevant mechanical signals to guide training and rehabilitation. In this review we provide a description of how wearable devices may be used in conjunction with computational rigid-body and continuum models of musculoskeletal tissues to produce real-time estimates of localized tissue stresses and strains. It is proposed that these bioinspired technologies will facilitate a new approach to physical training that promotes tissue strengthening and/or repair through optimal tissue loading.

## Introduction

Musculoskeletal diseases, such as osteoarthritis and tendinopathy, impose substantial burden on individuals and health care systems. As a community of scientists and clinicians, we have been largely ineffective in managing musculoskeletal diseases, as current prevalence, incidence, and socioeconomic burden are at alarming levels and projected to increase sharply in coming decades (Hunter et al., 2014). In particular, we have a limited understanding of how physical behavior, i.e., whole-body mechanics, influences tissue state (Forwood and Burr, 1993), and this could underpin our failure to cure, or curb, these prevalent, harmful, and costly diseases. A case in point is the study of the effects of physical activity on cartilage morphology. Studies of animals (Kiviranta et al., 1987, 1988, 1992; Newton et al., 1997) and humans (Jones et al., 2000, 2003; Roos and Dahlberg, 2005) have reported increased physical activity to be associated with positive structural and biochemical adaptations in weight-bearing joints, while other studies have reported no effects of physical activity on bulk measures of cartilage morphology (Eckstein et al., 2002, 2006).

The failure to effectively treat musculoskeletal disease is frustrating for scientists and clinicians alike. We possess a wealth epidemiologic data detailing risk factors for many musculoskeletal diseases, e.g., increased age, female sex, body mass, prior joint trauma, obesity, abnormal physical activity levels, and joint structural deformity (Felson et al., 1997, 2000, 2013; Cooper et al., 2000; Coggon et al., 2001; Lohmander et al., 2004, 2007; Roemer et al., 2009; Andriacchi et al., 2015). At tissue- and sub-tissue levels, studies have explored the effect of loading on structure and biology (Radin and Paul, 1971; Simon et al., 1972; Radin et al., 1973, 1984; Rubin and Lanyon, 1985; Forwood and Turner, 1995; Wang et al., 2013, 2015; Joo Kim et al., 2016). However, integrating experimental results with whole-body-, tissue-, and cell-level computational models, and using these models to modulate physical behavior to affect musculoskeletal tissue health remains challenging (Erdemir et al., 2015). In a recent narrative review, Ng et al. (2017) proposed physical therapy to enhance and promote tissue regeneration, linking external mechanical stimuli to tissue mechanobiology. In line with Ng et al. (2017), we describe an approach to deterministically quantify the link between physical behavior and tissue mechanobiology, inspired by integration of biomedical technologies (i.e., wearable devices, contemporary motion capture, and medical imaging) coupled to computational models of joints and musculoskeletal tissues.

Wearable body sensors and systems for “Quantified-Self” are set to transform how people interact with their environment and may facilitate personalized training and rehabilitation programs in the future. Biofeedback is a psychophysical process to augment awareness of afferent signals from sensory receptors in the human body. In the case of musculoskeletal tissues, biofeedback can be used to increase awareness and modify physical behavior (Sigrist et al., 2013). However, current rehabilitation and training protocols which incorporate biofeedback to modulate physical behavior target external biomechanics, such as the knee adduction moments (Barrios et al., 2010; Shull et al., 2011, 2013a,b; Wheeler et al., 2011) or gait spatiotemporal parameters (Wrigley et al., 2009; Erhart-Hledik et al., 2017). Eternal biomechanics are readily measured or calculated, and thus viable for use in biofeedback paradigms. Unfortunately, external biomechanics have tenuous relationships with internal biomechanics, such as articular contact loads (Walter et al., 2010; Winby et al., 2013; Saxby et al., 2016b).

**Figure 1:**
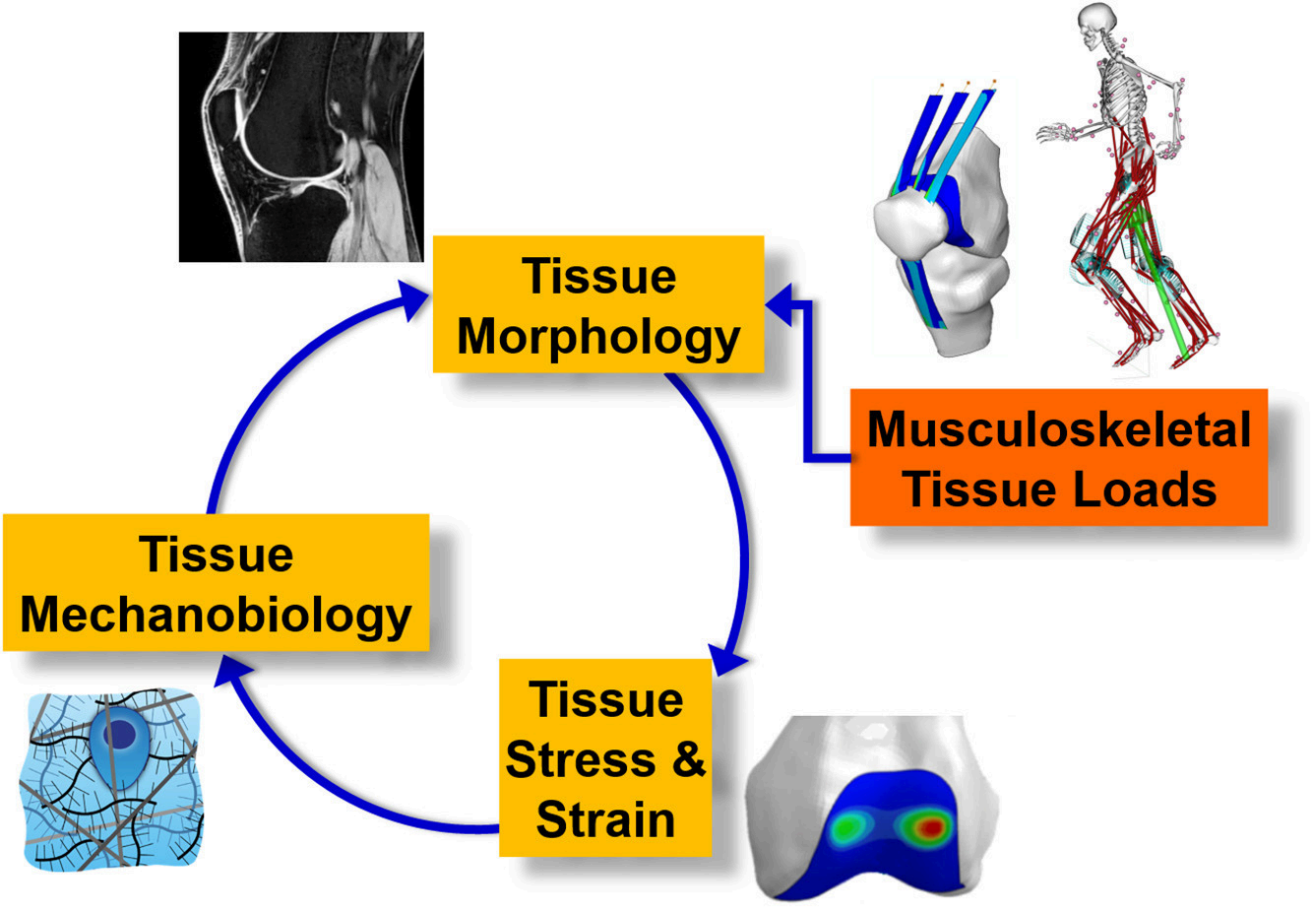
Schematic of complex dynamic interplay between external rigid body biomechanics, internal tissue biomechanics, tissue mechanobiology, and tissue state.

Musculoskeletal tissue stresses and strains are potentially superior to external biomechanics for use in biofeedback paradigms because they are physically coupled to the processes of mechanotransduction, whereby mechanical signals are registered as biologic stimuli, and result in cell- and tissue-level adaptations controlled by biologic regulatory mechanisms. However, musculoskeletal tissue stresses and strains have not been used in biofeedback technologies, because their computation is non-trivial, and depends on a complex interplay of multiple factors, including external biomechanics, neural control, tissue morphology and micro-architecture, and material properties (Figure 1). Importantly, recent advances in neuromusculoskeletal modeling have enabled real-time prediction of whole-body kinematics and external loading (Pizzolato et al., 2017a), as well as musculoskeletal tissue loading, such as muscle-tendon unit and articular contact forces during walking gait (Pizzolato et al., 2017b). Real-time musculoskeletal modeling can now be coupled to models of internal tissue mechanics and mechanobiology, and used to provide feedback to target training for tissue strengthening and repair.

In this narrative review we present an overview of (1) the known mechanical stimuli for promoting positive tissue adaptation in musculoskeletal tissues, (2) how local tissue stresses and strains can be estimated using computational methods, (3) an approach to estimating musculoskeletal tissue stresses/strains in real-time, and (4) challenges and future directions for research in this area.

## Mechanobiology and the optimal mechanical environment for musculoskeletal tissues

Mechanobiology is the study of the effect of mechanical stimuli on tissue biology. It is well-established that mechanical loading plays an essential role in (1) musculoskeletal tissue development throughout human maturation (Carter, 1987; Carter and Wong, 1988a,b, 1990; Wong and Carter, 1990; Carter et al., 1998, 2004; Beaupre et al., 2000), (2) maintenance of mature structures (Frost, 1988, 1990a,b,c,d), and (3) healing following injury, e.g., bone fracture (Pivonka and Dunstan, 2012). In particular, musculoskeletal tissues, such as articular cartilage, tendon, and bone, respond to strains by modulating tissue composition and organization. Generally, strains depend on the nature of applied loading, i.e., magnitude, location, orientation, duration, and frequency, as well as structural state of the object, i.e., morphology and material properties (Figure 1). Important to our study of musculoskeletal tissues, identical loads applied to different tissues (e.g., cartilage vs. bone vs. tendon), or same tissues but of different structural features (e.g., healthy vs. compromised, developing vs. mature), will produce different strains and eventually different biologic responses. Thus, to develop therapies targeting positive musculoskeletal tissue adaptations we must quantify relevant states. Equally important, if we wish evaluate therapeutic effectiveness we must also quantify changes to tissue states in response to those interventions.

### Estimating the state of musculoskeletal tissue

Musculoskeletal tissue state encompasses tissue morphology and function, both of which may be non-invasively assessed using medical imaging. Morphology, which encompasses all spatial descriptions of an object, can be measured using different medical imaging modalities, such as computed tomography (CT), magnetic resonance (MR), and ultrasound (US).

Computed tomography is well-suited to the study of bone and provides high-resolution images that can be automatically- or semi-automatically segmented to render volumetric representations (Dufresne, 1998). Peripheral quantitative CT can be used to image cortical and trabecular bone microstructure (Lespessailles et al., 2017), which are important structural features to include in analysis of bone remodeling (Hambli, 2011). However, CT exposes tissues to ionizing radiation and may not be suitable for certain clinical or developing populations.

Magnetic resonance imaging is a powerful modality that does not produce ionizing radiation, and can be used to image a wide range of musculoskeletal tissues (Hunter et al., 2015). However, individuals with implanted medical devices (e.g., cardiac stimulators) or ferrous prosthetics cannot safely undergo MR imaging. Unlike CT, MR images require manual segmentation to produce three-dimensional reconstructions of musculoskeletal tissues. Currently, manual segmentation is time consuming, but advances in image auto-segmentation (Mimics, Materialize NV, Leuven, Belgium) will hopefully reduce labor demands. Once MR images can be rapidly segmented, this will make MR imaging a routine process to assess musculoskeletal tissue morphology.

Ultrasound is an inexpensive, non-invasive, and non-radiating modality to image musculoskeletal tissues. Importantly, US can accurately measure muscle morphology (Barber et al., 2009), track muscle fascicles during contractions (Cronin et al., 2011; Gillett et al., 2013), and measure *in vivo* tendon morphology at rest and under load in healthy (Obst et al., 2014a,b) and pathologic tendon (Nuri et al., 2017). In addition to muscle-tendon applications, US has been used to measure bone landmark coordinates (Peters et al., 2010; Passmore and Sangeux, 2016) and make *in vivo* clinical measurements of bone alignment (Passmore et al., 2016). However, limited signal penetration into the body means that many deep anatomic structures cannot be imaged using US. Furthermore, deformation of soft tissues out of the imaging plane impairs measurement fidelity. To summarize, CT, MR, and US are imaging modalities capable of measuring musculoskeletal tissue morphology, however, morphology is only one component of tissue state, and alone is an insufficient indicator of tissue function and integrity.

Tissue function is related to tissue mechanical properties, such as stiffness and strength. As many pathologic tissue changes are accompanied by changes in tissue elasticity (Ophir et al., 1991), measures of tissue mechanical properties may serve as surrogate measures of tissue health and integrity. Henceforth, we will refer to medical imaging modalities used to assess musculoskeletal tissue mechanical properties as “functional imaging.” Elastography is a class of functional imaging, and is the study of elastic properties of materials. Elastography uses principles from the physics of wave propagation to quantify tissue mechanical properties (Ophir et al., 1991). In general, an internally- or externally- generated stimulus causes tissue deformation, which is measured and related to tissue elastic modulus (Yamakoshi et al., 1990). Relaxography is another class of functional imaging, whereby MR is used to indirectly assess tissue integrity by measuring time constants, e.g., T_2_, ${\text{T}}_{2}^{*}$, and T_1ρ_, associated with the slow motion of water molecules. Relaxography has emerged as a potent method to study and detect early signs of articular cartilage degeneration (Baum et al., 2013). As cartilage degenerates, its extracellular matrix is disrupted and proteoglycan content is reduced, which results in increased water content and motility. Relaxographic imaging is sensitive to early degenerative changes, as T_2_ relaxation times associated with healthy cartilages (~25–45 ms) are lower than those associated with degenerated cartilages (Dunn et al., 2004). Overall, there are several imaging modalities capable of assessing musculoskeletal tissue state, thus enabling creation of personalized musculoskeletal tissue models as well as quantifying intervention outcomes. However, it is first necessary to identify the optimal mechanical environments of each musculoskeletal tissue, which will serve as targets for bioinspired rehabilitation and training.

**Figure 2:**
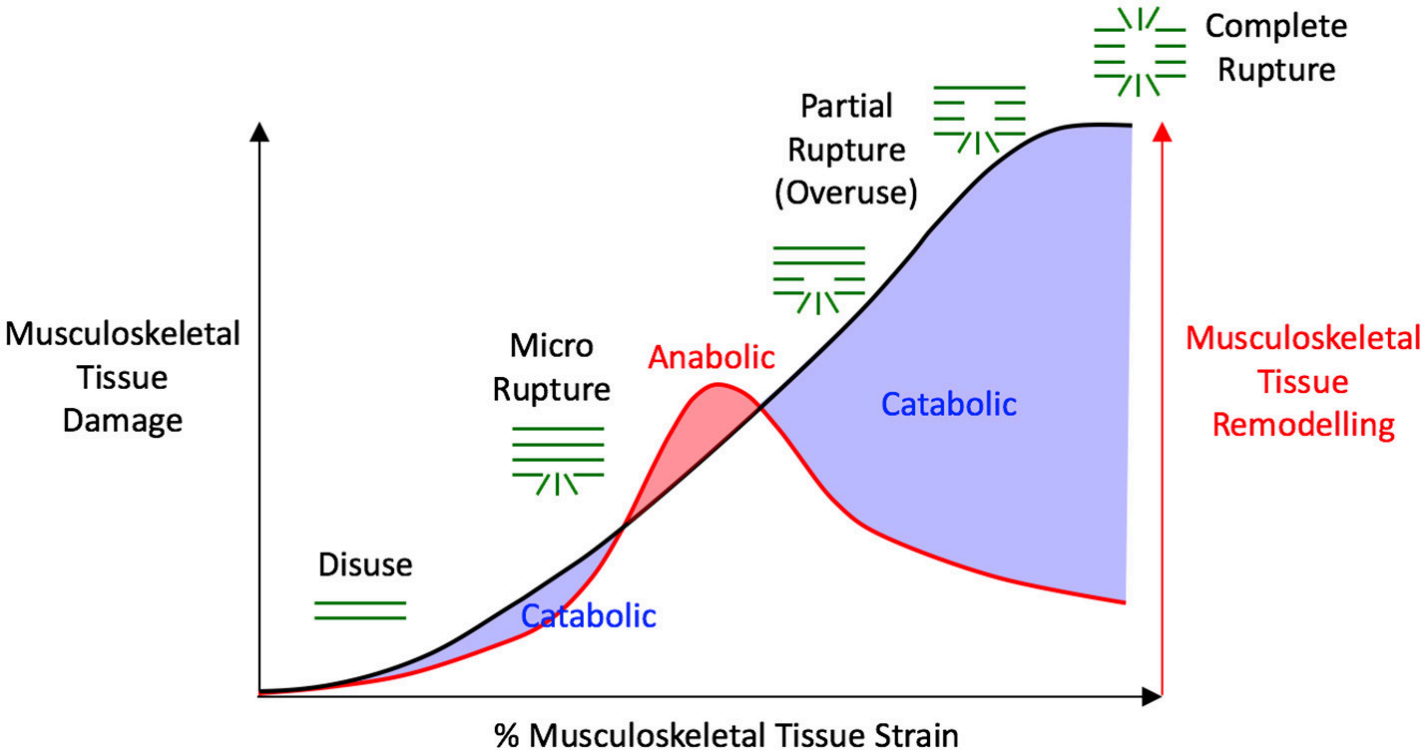
Schematic of mechanobiologic interplay between tissue strains that induce damage and remodeling. Within the anabolic “sweet spot” (i.e., red shaded area), tissues experience hypertrophy and improved mechanical properties. Within catabolic regions (i.e., two blue shaded areas), which are brought about due to over- or under-loading, tissues atrophy or degenerate, and this results in increased compliance and loss of strength. Adapted from Wang et al. (2013).

Just as hyper-physiologic loading can cause musculoskeletal tissue damage (Bonnevie et al., 2015; Christiansen et al., 2015), load deprivation due to low-gravity (Lang et al., 2006), or spinal-cord injury (Vanwanseele et al., 2002, 2003) causes tissue atrophy and weakening. More subtle changes in tissue loading can also affect tissue properties. For example a lower than normal knee contact force following orthopedic surgery has been associated with future onset of knee osteoarthritis (Wellsandt et al., 2016). Specifically, reductions of 10–20% of a body weight in the medial contact forces 6-months after anterior cruciate ligament reconstruction were associated with onset of medial knee osteoarthritis 5-years post-operation (Wellsandt et al., 2016). Similarly, animal experiments of unloading the weight-bearing limbs following knee ligament transection found subsequent muscle atrophy and loss of trabeculae (Anderson et al., 2016). Likewise, the human proximal tibia experiences substantial bone mineral density loss over the first year following anterior cruciate ligament reconstruction (Mundermann et al., 2015), which may be related to lower magnitude ambulatory tibiofemoral contact forces (Saxby et al., 2016a; Wellsandt et al., 2016). Overall, these results re-inforce the concept that inappropriate loading, due to over- and/or under-loading, precede articular tissue degeneration. It therefore follows that each tissue must have an optimal mechanical stimulus or “sweet spot” which maximizes anabolic tissue adaptation, where loads are neither too high to cause tissue damage, or too low to result in tissue degeneration (Figure 2).

### Tendon optimal mechanical environment

An illustrative example of a “sweet spot” in tissue regulation is drawn from *in vitro* studies of Achilles tendon. The Achilles tendon is a viscoelastic structure that links calf muscles, i.e., gastrocnemii and soleus, with the calcaneus bone of the foot, thus spanning the ankle joint. The Achilles tendon is crucial to common ambulatory tasks, such as walking, running, and jumping, through its role in biomechanical power generation and movement efficiency. When conditioned in a bioreactor, excised sections of Achilles tendons have shown optimal biomechanical response when subjected to ~6% cyclic tensile strains (Wang et al., 2013, 2015). Cyclic 6% tensile strains, 0.25 Hz loading cycle, 8 h per day, maintained tendon homeostasis (Wang et al., 2013) and, importantly, regenerated injured tendon (Wang et al., 2015). Consistent with the idea of over- and under-loading as mechanisms for tissue degeneration, tendon tensile strains below 3% or above 9% disrupted extracellular matrix (Wang et al., 2013), while tenocytes optimally responded to 4–6% strains (Joo Kim et al., 2016). These results reinforce the need to target specific strain ranges to maintain and repair tissue.

### Cartilage optimal mechanical environment

Articular cartilage caps the terminal regions of long bones involved in synovial joints, and provides a smooth ultra-low friction bearing surface for articulation. Articular cartilage is considered biphasic, consisting of a solid phase composed primarily of organized collagenous extracellular matrix interposed with chondrocytes, highly charged macromolecules, and immersed in an ionized interstitial fluid phase. Interaction between solid and fluid phases causes the mechanical behavior of cartilage, i.e., anisotropy, strength, and viscoelasticity (Mow et al., 1980; Armstrong and Mow, 1982). Indeed, the network of collagens and macromolecules of the extracellular matrix provide enormous resistance to internal fluid motility, primarily through friction. Consequently, during rapid loading of cartilage, as occurs during sport and activities of daily living, cartilage behaves as a nearly incompressible isotropic material. Internal resistance to fluid flow is an essential mechanism by which cartilage resists externally applied compression. However, as cartilage is avascular (Buckwalter, 2002; Buckwalter and Brown, 2004), large resistance to internal fluid flow prevents effective transport of materials and cells to injury sites. When cartilage deteriorates due to age, injury, or disease, it becomes more compliant (Setton et al., 1999). Consequently, collagen networks in the extracellular matrix experience larger, and potentially injurious, strains.

Limited interstitial motility combined with the avascular nature of cartilage, results in minimal regenerative capabilities (Newman, 1998; Buckwalter, 2002; Buckwalter and Brown, 2004). Understandably, research has focused on engineering cartilage implants and effective scaffolding to promote seamless uptake of implanted constructs into native cartilage. A recent review of literature found *in vitro* compressive strains of >20% applied at 0.5–1 Hz, were optimal for promoting cartilage cultivation (Natenstedt et al., 2015). A 20% strain is in the middle of the physiologic range experienced by cartilage (Grodzinsky et al., 2000), and 0.5–1 Hz loading rates are similar to the natural knee loading frequency during human gait. Thus, ~20% and 0.5–1 Hz cartilage strain and loading rate, respectively, provide logical targets to condition cartilage to prevent future degeneration, and may be effective to attenuate, stop, and even reverse degeneration in cases of established disease.

### Bone optimal mechanical environment

*In vitro* studies provide a rich source of direct measurements of strains that stimulate bone remodeling, as well as strains that injury or fracture bone. As many strain measures in the literature were acquired from experiments that did not incorporate bone dynamics, they should be considered time-independent mechanical targets, that may stimulate bone remodeling (Lanyon et al., 1975; O'connor et al., 1982; Rubin and Lanyon, 1985; Ehrlich and Lanyon, 2002). Different bone components, i.e., cancellous and cortical bone, may have different optimal strain ranges required to elicit adaptive remodeling. However, a range of ~200–1,000 με (1 με = 1 microstrain; 1 με = 0.0001% strain) represents everyday strains. During vigorous physical activities, such as sprinting, bone strains may reach peak values of ~2,000–3,000 με (Burr et al., 1996) and strain rates of ~10,000–50,000 μεs^−1^ (Lanyon et al., 1975). Even during vigorous physical activities, bone strains and strain rates do not necessarily damage tissue, as injurious strains are ~25,000 με in tension or compression directed longitudinally (Reilly and Burstein, 1974). Bone durability is first due to its innate capacity to withstand large stresses at low strain rates, i.e., ~80–170, ~100–300, and ~150–240 MPa in tensile, compressive, and bending modes, respectively (Reilly and Burstein, 1974). Second, bone is a viscoelastic material and its stiffness increases when subject to high strain rates, for example during running and jumping. Bone strains during strenuous physical activities have been reported to be ~10% of ultimate failure, well below bone fracture threshold and therefore considered safe healthy for individuals (Burr et al., 1996). Low impact and activities such as walking do not appear to be oesteogenic.

## Estimating the mechanical environment of musculoskeletal tissues

Musculoskeletal tissue state varies between individuals, and is affected by disease processes. To personalize therapy, we must account for subject-specificity, such that training programs can be tailored to the individual. A further technical challenge is that we need to estimate musculoskeletal tissue mechanics in real-time, providing an appropriate form of biofeedback to enable individuals to volitionally modulate tissue mechanics during rehabilitation, recreation, or daily activities. To achieve this goal we must merge whole-body representations of human physical behavior with models of musculoskeletal tissue mechanics and mechanobiology within efficient computational frameworks.

Currently, there is no feasible method to directly measure *in vivo* loading applied to, and stresses/strains within, musculoskeletal tissues in *native* human joints. Articular contact forces can be measured in cadavers through a combination of robotic control and mathematical modeling (Wang et al., 2014) or by inserting pressure sensitive film between articulating surfaces (Ihn et al., 1993). However, a valid method of applying physiologic muscle, body, and inertial loads to cadavers has not been reported, thus casting doubt whether these measurements are representative of *in vivo* loading. Contact forces can also be measured by instrumenting prostheses used in arthroplasty, as has been done at knee (D'lima et al., 2005, 2006; Heinlein et al., 2007, 2009; Fregly et al., 2012), hip (Rydell, 1966; English and Kilvington, 1979; Bergmann et al., 2010), and shoulder (Bergmann et al., 2007) joints. Contact loads measured by instrumented prostheses provide critical information to implant designers regarding the nature of the mechanical demands placed upon these devices. Unfortunately, instrumented prosthetic implants are only appropriate for measuring contact loads in arthroplasty patients, who are typically elderly individuals with substantially degenerated joints and peri-articular muscle atrophy. Furthermore, arthroplasty, by definition, does not preserve the native joint and restricts the activity types that could be studied in these patients, e.g., it is unethical to ask an elderly knee arthroplasty patient to perform vigorous athletic movements. Consequently, contact loads sustained by implants are unlikely to be representative of contact loads in native joints of young physically active populations.

In addition to articular contact forces, muscle-tendon unit forces have also been directly measured in both animals (Walmsley et al., 1978; Hodgson, 1983; Herzog et al., 1992) and humans (Gregor et al., 1987; Komi et al., 1987; Fukashiro et al., 1995) by surgically implanting mechanical gauges. Proficient surgical implantation results in minimal inflammatory response, and instruments may left *in situ* in animals for days or even weeks. However, extrapolating *in vivo* animal muscle-tendon force measurements to humans is questionable and certainly of limited clinical relevance. In humans, surgical implantation of strain gauges into tendon may affect an individual's physical behavior, thus limiting ecologic validity of the measurements. Furthermore, muscle-tendon forces are subject-, task-, and state-specific, thus limiting applicability of measurements from an individual performing a specific task to another individual, movement or control task, or the same individual at a later date, i.e., following an intervention, suffering an injury, or onset of disease. Given the serious limitations of direct measurement of musculoskeletal tissue loads, researchers have used computational neuromusculoskeletal to predict musculoskeletal tissue loading.

### Neuromusculoskeletal models to estimate musculoskeletal tissue loading

Neuromusculoskeletal models are bioinspired mathematical representations of specific neurologic, physiologic, and anatomic characteristics of an individual (Hatze, 1977; Buchanan et al., 2004, 2005; Lloyd et al., 2005). Neuromusculoskeletal models may be used to estimate muscle (Lloyd and Besier, 2003; Erdemir et al., 2007), ligament (Shelburne and Pandy, 1997; Pandy and Sasaki, 1998; Lloyd et al., 2005; Shelburne et al., 2005), and articular contact forces (Shelburne et al., 2005; Winby et al., 2009; Gerus et al., 2013; Manal and Buchanan, 2013; Erdemir et al., 2015; Walter et al., 2015; Saxby et al., 2016b; Smith et al., 2016; Konrath et al., 2017), and have been deployed across a wide range of scientific, industrial, and clinical applications, such as investigating fundamental properties of human motor control (Haeufle et al., 2014; Sartori et al., 2015), evaluating ergonomic demands of automotive operation (Rasmussen et al., 2009), and informing medical device designs by predicting *in vivo* loading conditions (Marra et al., 2015). Typically, structural characteristics used in a model are based on measurements from a small number of cadavers, and subsequently used as a generic template for each analysis. Bone dimensions and mass-inertia properties in a generic template are linearly scaled to match subject's dimensions (Delp et al., 1990), thus providing a basic level of model personalization. Using generic templates facilitates rapid and routine use of neuromusculoskeletal models, but scaled generic models are often poor representations of an individual's musculoskeletal anatomy, which may lead to inaccurate results, spurious conclusions, and potentially detrimental clinical decisions. For example, linear scaling of a generic model template may result in incorrect representation of muscle moment arms (Arnold et al., 2000; Scheys et al., 2008) and consequently erroneous joint contact force estimates (Lenaerts et al., 2009; Gerus et al., 2013; Wesseling et al., 2016).

Several aspects of neuromusculoskeletal models can be personalized to the individual to improve simulation results. Bone morphology and joint mechanics have been shown to influence kinematics and kinetics estimates (Brito da Luz et al., 2016; Kainz et al., 2016), and knee contact forces have been shown to be sensitive to tibiofemoral alignment (Lerner et al., 2015). Skeletal geometry also affects muscle-tendon paths and insertion points, which in turn define muscle-tendon lines of action, influencing both muscle-tendon lengths and moment arms. Overall, better representation of an individual's musculoskeletal anatomy has been shown to produce more realistic results, e.g., improved representation of muscle-tendon moment arms, improved knee (Gerus et al., 2013) and hip (Modenese et al., 2013) contact forces estimates.

Muscle activation patterns are known to vary between individuals and controls tasks (Tax et al., 1990; Buchanan and Lloyd, 1995), and are affected by training (Menegaldo and Oliveira, 2011) and pathology (Besier et al., 2009). Incorporating experimental measures of muscle activation patterns into neuromusculoskeletal models adds an important dimension of personalization. Electromyography (EMG)-informed neuromusculoskeletal models (Manal et al., 2002; Lloyd and Besier, 2003; Manal and Buchanan, 2013; Sartori et al., 2014; Pizzolato et al., 2015) are a class of neuromusculoskeletal models sensitive to variations in motor control. Specifically, EMG-informed neuromusculoskeletal models use experimentally measured muscle excitations and movement patterns to account for complex interplay between external biomechanics and muscle recruitment to estimate musculoskeletal tissue loadings, i.e., joint, muscular, ligamentous, and articular contact loads, that may serve as boundary conditions for continuum mechanics analysis.

### Finite element method to estimate musculoskeletal tissues mechanical environment

The finite element method (FEM) is a well-established computational method used in many branches of engineering. In a FEM model, the real system is discretized into a field of elements of known geometries and material properties, from which constitutive equations may be developed. The model system dynamics are then equilibrated by imposing a set of boundary conditions, e.g., musculoskeletal tissue loads informed by a neuromusculoskeletal model. Halloran et al. (2010) applied this combined neuromusculoskeletal and FEM modeling to foot and ankle strains, while Besier et al. (2009) verified predicted patellofemoral stresses/strains using measurements of cartilage deformation acquired in a vertical bore MR unit. Recently, others have explored tibiofemoral cartilage stresses/strains during gait (Shim et al., 2016; Smith et al., 2016) and acetabulum stress distributions in relation to bone remodeling (Fernandez et al., 2014). These studies have shown the potential of the FEM models, but the models employed were not fully personalized.

Generating personalized FEM models of tissue requires both morphology and material properties. As previously described, different imaging modalities can be used to directly acquire tissue-specific morphology, but non-invasive methods to estimate material properties are scarce. Musculoskeletal tissues have a heterogeneous multiphasic structure, resulting in anisotropies and non-linear time-varying behavior (Freutel et al., 2014), thus making the estimation of material properties challenging. Relaxography (Labadie et al., 1994) is a powerful tool to assess tissue function, but it only provides measures which correlate with, but do not quantify, tissue material properties (Lammentausta et al., 2006). Elastography (Ophir et al., 1991) can provide direct measurement of musculoskeletal tissue stiffness by analyzing the response of tissue to external stimuli. In MR elastography, low frequency vibrations are externally introduced to the body by means of electromechanical devices, while multiple images are recorded to analyse tissue response at different time points and directions (Glaser et al., 2012). Ultrasound elastography works by the same principle as MR elastography, but the external stimuli can be provided by the US transducer itself. Ultrasound-based shear-wave elastography has recently been applied to musculoskeletal tissues (Eby et al., 2013) to quantify stiffness, but is limited to superficial tissues and subject to errors due to probe positioning (Brandenburg et al., 2014). Reverse FEM could also be used to estimate tissue material properties, whereby tissue is subject to multiple and explicitly known applied loading conditions that alter morphology. A numerical optimization then estimates a set of material properties best fitting the measured morphology change (Hansen et al., 2017).

Informing tissue material properties in FEM models through non-invasive imaging would provide a level of personalization well beyond current standard approaches, which typically apply literature values established through experiments performed on cadavers. Indeed, tissue material properties are specific to individuals and are affected by aging, training, injury, and disease (Arokoski et al., 2000; Buckwalter, 2002; Buckwalter and Brown, 2004). Different tissue stress and strain patterns will arise from FEM simulations that use different tissue material properties, even when composed of identical tissue morphology and subjected to identical boundary conditions. Finally, simulations of musculoskeletal tissue mechanics may use physiologic and personalized boundary conditions informed by neuromusculoskeletal models (Besier et al., 2005, 2009; Fernandez et al., 2014). Overall, when FEM models of musculoskeletal tissues are informed by measurements of subject-specific morphology, material properties, and boundary conditions, they are powerful tools to understand musculoskeletal tissue mechanics.

### Finite element method to estimate musculoskeletal tissue remodeling

Considerable research focus has been applied to studying relationships between applied tissue loading and morphology, with a fundamental assumption that tissue health may be assessed through structural analysis (e.g., thicker cartilage is indicative of healthy cartilage; Koo and Andriacchi, 2007). Rigid-body computational models have been used to determine external joint or articular loads, which in turn have been compared to measures of articular tissue structure using linear statistics (Koo and Andriacchi, 2007; Koo et al., 2011; Scanlan et al., 2013; Van Rossom et al., 2017). However, primary focus on applied loading may not be appropriate, as other biomechanical signals, such as extracellular fluid motion in bone (Zadpoor, 2013; Villette and Phillips, 2016) or bone strain energy (Kerner et al., 1999), are physically closer to cellular mechanisms of remodeling and have been shown to influence tissue adaptation. Simulations of trabecular remodeling have been performed whereby structural modifications were driven by local mechanical criteria, e.g., minimizing density of material anisotropy with respect to principle stresses (Fyhrie and Carter, 1986) or non-uniformity in local stresses (Adachi et al., 1997; Tsubota et al., 2002). Such simulations were able predict trabecular distributions consistent with experimental observations (Fyhrie and Carter, 1990), and results were highly sensitive to loading condition complexity. When complex loading patterns were applied to FEM models with embedded bone remodeling algorithms, predicted bone material property distributions were consistent with *ex vivo* imaging (Geraldes et al., 2016). Similarly, features such as bone cortical thickness and regional femoral trabecular density were better predicted when complex physiologic loads were applied compared to simple axially oriented compressive loads (Geraldes et al., 2016). When complex muscle loading patterns were included in FEM simulations of femoral bone remodeling in the context of prosthetic hip implants, simulations predicted bone retention patterns in regions of muscle attachment, which is not predicted by FEM models using simple idealized boundary conditions (Bitsakos et al., 2005). These results suggest incorporation of complex biomechanical loads into FEM models is required to predict correct spatial distribution and peculiar features of musculoskeletal tissue structure.

The complex biomechanical loads sustained by the human body are generated by non-linear muscle dynamics and their interaction with convoluted three-dimensional musculoskeletal architecture. Including muscle dynamics into FEM models directly affects spatial distribution of musculoskeletal tissue strains, and hence influences predictions of tissue remodeling (Duda et al., 1998). When pairing together computational rigid-body neuromusculoskeletal and FEM models, the degrees of freedom associated with the respective models must be consistent (Phillips et al., 2015). For example, the popular musculoskeletal modeling software OpenSim (Delp et al., 2007) enables users to define complex joint motions that are both arbitrarily bounded and computationally efficient (Seth et al., 2010). The OpenSim model may then be used to solve external joint and muscle loads, which can, in principle, be applied to FEM models. However, the FEM model must be constrained in an analogous manner to the OpenSim model to ensure model degree of freedom consistency. This is not a peculiar consideration of OpenSim models, rather, all hierarchical modeling frameworks which combine boundary conditions from a rigid-body simulation to a FEM model should respect this demand for consistency. In the context of bone remodeling simulations, Phillips et al. (2015) presented a method to ensure model degree of freedom consistency, but noted that the constraints of model displacement may limit scope of the analysis.

**Figure 3:**
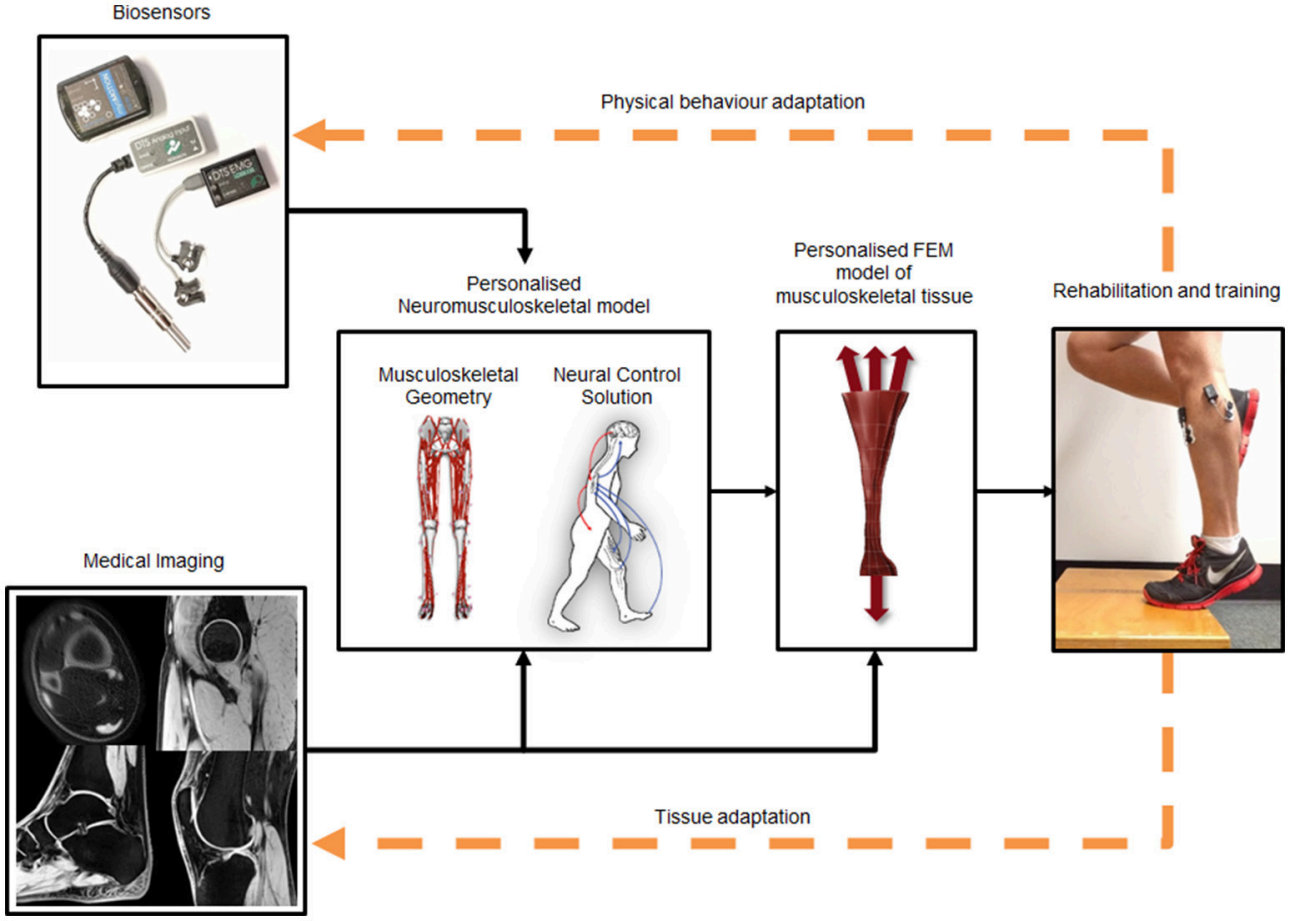
Framework to estimate *in vivo* musculoskeletal tissue stresses and strains. Medical imaging is used to create personalized musculoskeletal geometry and FEM models of the tissue of interest. Biosensors (e.g., EMG, inertial measurement units, and/or motion capture) are used to drive a neuromusculoskeletal model, which provides boundary conditions necessary for the FEM model to estimate musculoskeletal tissue stresses and strains. Tissue stresses and strains can be fed-back, in real-time, to enable the person to modify their behavior to affect tissue mechanical environment. Finally, tissue and physical behavior adaptations update the computational system indicated by orange dashed feedback arrows.

Optimal mechanical environments for musculoskeletal tissue adaptation have been provided from *ex vivo, in vitro*, and *in silico* studies. This knowledge, combined with an appreciation for modeling complexity required to estimate musculoskeletal tissue stresses and strains, leaves us well posited to move forward and apply these models in clinical contexts such as training or rehabilitation. If we can gain control of an individual's physical behavior through biofeedback paradigms, and target the optimal *in vivo* mechanical environment of their musculoskeletal tissues, we may be able to prevent tissue deterioration or restore health. We represent our vision in Figure 3, and its realization would be a breakthrough for rehabilitation science and medicine. However, to realize this aim, current computation processes that are performed offline must be performed in real-time.

## Real-time estimation and biofeedback of musculoskeletal tissue stress and strain

Behavioral movement changes, in the form of modulation of body kinematics and kinetics, have been used in rehabilitation to assist motor learning and improve function following injury or disease (Sigrist et al., 2013). Much research has focused on biofeedback technologies to improve movement and function in knee osteoarthritis patients (Barrios et al., 2010; Shull et al., 2013a,b; Van Den Noort et al., 2015). In these patients, larger magnitude walking knee adduction moments have been associated with structural progression of knee osteoarthritis (i.e., joint space narrowing; Miyazaki et al., 2002) and knee pain (Amin et al., 2004), making reduction of the magnitude of the knee adduction moment a logical target for physical therapy.

Numerous studies have combined biofeedback technologies with gait modification strategies to modify joint kinematics or external loads with the aim of improving health outcomes or reducing movement variability. For example, real-time visual biofeedback of upper-body posture (Hunt et al., 2011) and dynamic knee alignment (Barrios et al., 2010) have been used to reduce walking knee adduction moments in healthy individuals and knee osteoarthritis patients, respectively. Knee braces instrumented with auditory feedback have been used to reduce knee loading rates during walking (Riskowski et al., 2009), while instrumented footwear has been used to reduce lateral foot pressures through vibrotactile feedback (Dowling et al., 2010). Notably, Shull et al. (2013b) provided haptic feedback, delivered through body worn vibrating motors, to inform participants of their changes in foot progression and trunk sway during treadmill walking. This resulted in patients with knee osteoarthritis reducing their peak knee adduction moment magnitudes. However, the main limitation of modifying external kinematics and kinetics is their tenuous relationships with internal loads (Walter et al., 2010, 2015; Winby et al., 2013; Saxby et al., 2016b), which implies weaker still relationships to articular tissue stresses and strains. The reason for these poor relationships is external biomechanics cannot account for the direct effect of muscles on musculoskeletal tissue loading (Walter et al., 2010; Winby et al., 2013; Saxby et al., 2016b).

As previously discussed, neuromusculoskeletal models can provide FEM with appropriate boundary conditions to estimate musculoskeletal tissue stresses and strains (Besier et al., 2005, 2009; Fernandez et al., 2014). This may be done in an offline analysis, but real-time estimation of musculoskeletal tissue stresses and strains requires interfacing with, and enabling data flow from, external devices to modeling software to complete necessary computations within given time constraints. For neuromusculoskeletal models, this means solving kinematics, kinetics, and muscletendon forces in real-time. Muscle forces have been calculated in real-time using a static optimization method (van den Bogert et al., 2013), where an algorithm determined the minimized weighted sum of muscle forces that matched external joint moments (Van Der Helm, 1994). However, the real-time approach presented by (van den Bogert et al., 2013) was based on a generic anatomic model that could not be personalized. Model personalization, noted earlier in this review, is essential when coupling neuromusculoskeletal and FEM models of musculoskeletal tissue mechanics. Furthermore, many neuromusculoskeletal models rely on mechanical optimization to solve the muscle redundancy problem (Crowninshield, 1979; Crowninshield and Brand, 1981), however, mechanical optimization methods struggle to predict many empirical features of muscle coordination, such as patterns of muscle activation (Herzog and Binding, 1992), co-contraction (Herzog and Binding, 1993), and force sharing (Binding et al., 2000). To our knowledge, the first use of a real-time EMG-informed neuromusculoskeletal model was by Manal et al. (2002), and first applied to musculoskeletal tissue loading in Achilles tendon rehabilitation by Manal et al. (2012). These papers advanced the field and should be acknowledged as pioneering, but were limited in application to quasi-static movements and a single joint with few degrees of freedom. Recently, Pizzolato et al. (2017b) developed software, based on OpenSim (Delp et al., 2007), to calculate full-body kinematics and kinetics (Pizzolato et al., 2017a), as well as musculoskeletal tissue loading (Pizzolato et al., 2015, 2017b), in real-time. Their method is fully extensible to other joints and musculoskeletal tissues, but is currently limited to expensive and immobile laboratory-based stereophotogrammetry systems (Pizzolato et al., 2017b).

Wearable sensors that accurately estimate human kinematics are a promising alternative to laboratory-based measurement systems. Linear accelerometers have been used for many years to quantify movement patterns relative to the gravitational field and ambulatory temporal-spatial parameters (Kavanagh and Menz, 2008), but their estimates of joint kinematics are limited by signal drift caused by integration errors (Djuric-Jovicic et al., 2011). Improvements in microelectromechanical systems have enabled embedding tri-axial accelerometer, gyroscope, and magnetometer into a single sensor. These integrated sensors are known as inertial measurement units and are able to estimate spatial orientation (Sabatini, 2006; Madgwick et al., 2011) and, when used in combination with anatomic models, joint angles. Strain sensors are another class of promising wearable sensors that can be used to estimate joint angles (Nakamoto et al., 2016). Strain sensors are low profile, flexible, and can be easily embedded into garments or mounted on the skin (Amjadi et al., 2016). To date, strain sensors have been used in biomechanics primarily to classify movement (Mattmann et al., 2007) or estimate single joints angles (Nakamoto et al., 2016). However, continuous technologic improvements in smart textiles (Honarvar and Latifi, 2017) may soon lead to advanced garments capable of estimating full-body kinematics.

Measuring or estimating reaction forces between body and ground is required to correctly estimate load applied to specific musculoskeletal structures, such as joints and ligaments. In laboratory conditions, ground reaction forces are acquired via ground mounted force plates, but alternative solutions are required for applications in the real-world. Pressure-sensitive insoles can be used to estimate the normal component of the ground reaction force, but they neglect shear components (Chesnin et al., 2000). Conversely, shoes instrumented with tri-axial force sensors have shown agreement with force plates for all components of the ground reaction force vector (Liedtke et al., 2007). Alternative to measurements, deep learning algorithms have been shown to correctly estimate ground reaction forces during walking (Oh et al., 2013). However, these data-driven models require big data as training sets. Mechanical approaches can be used to solve dynamics of motion and estimate ground reaction forces without body-worn force sensors. For example, the zero-point moment is an algorithm developed for humanoid robots (Xiang et al., 2009) that has also been successfully applied human biomechanics (Fluit et al., 2014; Dijkstra and Gutierrez-Farewik, 2015). However, to correctly estimate ground reaction forces, full-body kinematics and subject-specific musculoskeletal models are required (Fluit et al., 2014).

Overall, advances in wearable sensors, i.e., smaller, lighter, low-power, and integrated sensor systems, will enable novel real-world applications (Brodie et al., 2008). Currently, intrinsic limitations and measurement inaccuracies associated with these devices prevent their use in advanced biomechanical analysis. Combining wearable sensors with sophisticated biomechanical models may help to minimize the limitations associated with wearable sensors. Realistic musculoskeletal models, such as those offered by OpenSim (Delp et al., 2007), associated with probabilistic frameworks that adequately model wearable sensor inaccuracies (Latella et al., 2016) and computationally efficient real-time software architectures (Pizzolato et al., 2017a,b), have the potential to accurately estimate human motion in real-world setting free from the laboratory.

As previously stated, boundary conditions for subsequent FEM model simulations may be computed in real-time using neuromusculoskeletal models. However, even if appropriate boundary conditions are provided, real-time solutions to continuum mechanics problems is an ongoing computational challenge. When implementing entire musculoskeletal structures in FEM models (e.g., complete bones), computational demand may be substantially reduced by spatially averaging many microstructural features, such as trabecular and cortical bone architecture. However, spatial averaging neglects analysis of tissue anisotropy and micro-architecture, which are known to influence tissue function (Stein et al., 2010). Generally, FEM models are computationally demanding and not solvable in real-time. Thus, FEM models must be reduced to surrogates by a process known as “Kriging” (Matheron, 1963), whereby the continuum model is first solved offline for all possible, or physiologic, configurations (Wu et al., 2014; Eskinazi and Fregly, 2016), and simulation results are then be stored for future real-time use. However, it is computationally expensive to establish robust surrogates of musculoskeletal tissue continuum models, given the large data throughput imposed by performing many multi-scale simulations (Erdemir et al., 2015). One potential strategy we are pursuing is use of high-performance computing, whereby a large number of simulations are managed and solved by a remote computing cluster.

## Challenges and future directions

Our proposed framework to modify an individual's physical behavior to optimize musculoskeletal tissue mechanobiology (Figure 3) is feasible and currently being developed. In Table 1 we have summarized several challenges and possible future directions discussed in the text. To move these bioinspired technologies to clinical settings we need to direct our efforts toward: (1) rapid generation and seamless integration of personalized neuromusculoskeletal and FEM models, (2) use of wearable sensors, and (3) improvement of biofeedback modalities for stress and strain modulation.

**Table 1:** Summary of the various challenges faced in modeling tissue mechanobiology and using biofeedback to modulate *in vivo* tissue strains in real-time.

**Area**	**Challenge**	**Possible solution**
Mechanobiology	Validating *in vitro* and *in silico* estimates of optimal remodeling strains	Targeted mechanobiology experiments in bioreactor
Neuromusculoskeletal models	Rapid generation of personalized models	Rapid autosegmentation of medical imaging
		Statistical shape modeling based on large medical imaging databased
FEM models	*In vivo*, non-invasive, accurate determination of material properties	Advancements in elastography and relaxography methods
		Numerical optimization via reverse FEM
	Real-time evaluation	Surrogate models
		High performance computing
	Generation of robust surrogates of continuum models	Open challenge
Wearable biosensors	Measuring body motion, loading, muscle activation out of the laboratory	Wearable biosensors embedded in garments
		Reducing the number of required sensors
	Accurate kinematics estimation	Inertial measurement units or strain sensors coupled with accurate anatomic models and probabilistic frameworks
	Accurate kinetics estimation	Deep learning algorithms and training databases
		Zero-point moment algorithms coupled with optimization, deep learning, or pressure sensors to solve for double stance
		Instrumented shoes
Biofeedback	Establishing effective biofeedback variable	Processing tissue strain using mechanoreceptors transfer functions
Clinical translation	Seamless technology simple to use	Target specific tissues to reducing the number of sensors and details of models
		Analyse the effect of model simplifications on tissue strain prediction

Currently, creating personalized anatomic models from medical imaging is expensive (i.e., involves costly image acquisition and numerous man-hours to process raw medical imaging into high-fidelity computational models). However, improvements in image processing software, such as automatic segmentation and statistical shape modeling (Zhang et al., 2014; Zhang and Besier, 2017) may greatly accelerate model generation. Statistical shape modeling is promising as it may limit the need to acquire expensive medical imaging, relying instead upon a musculoskeletal atlas database to characterize an individual's anatomy from sparse or meta-data (Zhang and Besier, 2017). However, it is unclear whether current publically available medical imaging databases are sufficient to represent the variability in musculoskeletal anatomy in clinical populations, or those with traumas or implants. This is a limitation that will eventually be addressed by data sharing amongst research teams, which is an effort we thoroughly support.

Another limitation is that motion capture systems typically used in research gait laboratories are seldom used into clinical settings, because of their complexity, space requirements, and high purchase and operational costs. For bioinspired technologies to be broadly adopted, we need to free ourselves of traditional motion capture systems and look to wearable sensors to measure movement, external loads, and muscle excitation. A promising and relatively inexpensive example of wearable sensors that could help us on this mission are inertial measurement units, which provide a wealth of data that may be used to determine whole-body motion. Currently, inertial measurement units are limited by issues such as insufficient shielding from electromagnetic interference (i.e., while walking on treadmills or near informatics cabling) and registration of body-worn sensor positions to anatomic models. Wireless EMG systems have been used effectively in research and clinical settings for many years, and are now being integrated with inertial measurement units and other sensors as standalone devices or embedded into garments. Future research should aim to first establish if these wearable sensor systems can match the performance of traditional motion capture systems, and then minimize the number of sensors required to drive neuromusculoskeletal models.

Very little is currently known about the ability of individuals to volitionally modulate musculoskeletal tissue stresses and strains in response to real-time feedback. To our knowledge, musculoskeletal tissue stresses and strains have never been estimated in real-time, let alone used to modify physical behavior. To date, only two research groups (Manal et al., 2012; Pizzolato et al., 2017b) have provided real-time biofeedback of musculoskeletal tissue loads, but their work has been limited to muscle-tendon and rigid articular contact forces, and did not model tissue stresses and strains.

We know from previous studies people can use visual biofeedback to manipulate external biomechanical variables, muscle excitations, and tibiofemoral contact forces (Manal et al., 2012; Pizzolato et al., 2017b). Future research should strive to identify the biofeedback modality optimal for modulating musculoskeletal tissue stresses and strains through changes in human movement and muscle activation. Further, it may be possible to draw inspiration from a variety of native mechanoreceptors in the human body to provide enhanced visual biofeedback of stresses and strains. We imagine a technology whereby estimates of musculoskeletal tissue loading (i.e., forces or stresses and strains) could be transformed according to golgi organelle and muscle spindle transfer functions to provide more intuitive biofeedback.

By optimizing the mechanical environment it may be possible to regulate musculoskeletal tissue mechanobiology, potentially preventing disease, or restoring degraded tissue to health. Consequently, modeling and controlling physical behavior of individuals has enormous implications for development and management of chronic musculoskeletal diseases such as osteoarthritis or tendinopathies. We have presented a framework to move from *in vitro* and *ex vivo* studies of tissue mechanobiology to personalized *in silico* real-time models of musculoskeletal tissue loading. Integrating and translating these bioinspired technologies to clinical settings will prove challenging and resource intensive. Skepticism from clinicians accustomed to generic recommendations based on linear statistics is anticipated and will need to be overcome by demonstrating the efficacy and clinical utility of the proposed new approach. However, there awaits a wide spectrum of important clinical conditions to which these bioinspired technologies could be applied with the goal of reducing the socio-economic burden of musculoskeletal diseases.

## Author contributions

CP, DL, and DS contributed to conceptualize, draft, critically revise, and approve the final version of the article. RB, JC, MZ, and TB contributed to conceptualize, critically revise, and approve the final version of the article.

### Conflict of interest statement

The authors declare that the research was conducted in the absence of any commercial or financial relationships that could be construed as a potential conflict of interest.
